# Motor Efficacy of Subcutaneous DIZ102, Intravenous DIZ101 or Intestinal Levodopa/Carbidopa Infusion

**DOI:** 10.1002/mdc3.14138

**Published:** 2024-06-24

**Authors:** Filip Bergquist, Mats Ehrnebo, Dag Nyholm, Anders Johansson, Fredrik Lundin, Per Odin, Per Svenningsson, Nil Dizdar, Elias Eriksson

**Affiliations:** ^1^ Department of Pharmacology University of Gothenburg Gothenburg Sweden; ^2^ Sahlgrenska University Hospital Gothenburg Sweden; ^3^ Department of Pharmaceutical Biosciences Uppsala University Uppsala Sweden; ^4^ Ehrnebo Development AB Uppsala Sweden; ^5^ Department of Medical Sciences, Neurology Uppsala University Uppsala Sweden; ^6^ Department of Clinical Neurosciences Karolinska Institutet Stockholm Sweden; ^7^ Department of Biomedical and Clinical Sciences Linköping University Linköping Sweden; ^8^ Division of Neurology, Department of Clinical Sciences Lund University Lund Sweden

**Keywords:** carbidopa, levodopa, continuous subcutaneous infusion, Parkinson's disease

## Abstract

**Background:**

It has been suggested that carbidopa at high blood concentrations may counter the therapeutic effect of levodopa in Parkinson's disease by entering the brain and blocking central levodopa conversion to dopamine. We previously demonstrated equivalent plasma levodopa concentration in patients with Parkinson's disease during 16 h of (1) intravenous carbidopa/levodopa (DIZ101) infusion, (2) subcutaneous carbidopa/levodopa (DIZ102) infusion or (3) intestinal carbidopa/levodopa gel infusion. Plasma levels of carbidopa were however approximately four times higher with DIZ101 and DIZ102 than with LCIG, and higher than those usually observed with oral levodopa/carbidopa.

**Objectives:**

To investigate if high carbidopa blood concentrations obtained with parenteral levodopa/carbidopa (ratio 8:1) counter the effect of levodopa on motor symptoms.

**Methods:**

Eighteen patients with advanced Parkinson's disease were administered DIZ101, DIZ102, and intestinal levodopa/carbidopa gel for 16 h on different days in randomized order. Video recordings of a subset of the motor examination in the Unified Parkinson's Disease Rating Scale (UPDRS) were evaluated by raters blinded for treatment and time. Motor function was also measured using a wrist‐worn device monitoring bradykinesia, dyskinesia, and tremor (Parkinson KinetiGraph).

**Results:**

There was no tendency for poorer levodopa effect with DIZ101 or DIZ102 as compared to LCIG.

**Conclusion:**

Although DIZ101 or DIZ102 causes approximately four times higher plasma carbidopa levels than LCIG, patients responded equally well to all treatments. The results do not indicate that high plasma carbidopa levels hamper the motor efficacy of levodopa.

Substitution of dopamine with the dopamine precursor levodopa and a peripheral decarboxylase inhibitor, sometimes combined with a catechol‐O‐methyltransferase inhibitor, remains the most efficacious pharmacological treatment for Parkinson's disease (PD).^1^ However, with progressing disease, this treatment is commonly associated with fluctuations in motor function, expressed as hypokinesia when the dopamine concentration is low and involuntary hyperkinetic movements when it is high or changing (on–off).^2^ The importance of fluctuations in levodopa blood concentrations for these motor fluctuations was established already in the 1970s by the demonstration of significant improvement when oral treatment was replaced by an intravenous infusion.^3^

Continuous levodopa infusion was first established as clinical treatment in the late 1990s, when the problem of low levodopa solubility at physiological pH was circumvented by the introduction of a carbidopa/levodopa‐containing gel pumped continually into the intestines via a percutaneous gastro‐jejunal tube (LCIG; Duodopa®/Duopa®).^4^ The superiority of this treatment over oral levodopa/carbidopa administration with respect to daily time with good function (on‐time without disturbing dyskinesias) has been convincingly confirmed providing clinical proof of concept for the superiority of continuous over intermittent levodopa administration in outpatients with advanced PD.^5^, ^6^ More recently, concentrated levodopa/carbidopa preparations for subcutaneous use, ND061 and ABBV‐951, have been shown to produce robust increases in on‐time without disturbing dyskinesia.^7^, ^8^

DIZ102 is another concentrated carbidopa/levodopa solution for subcutaneous administration and consists of a low pH stock solution with levodopa and carbidopa that is brought to a physiologically favorable pH (5.0–5.3) by continuous mixing with a buffer solution during infusion. DIZ101 is a similar product but intended for intravenous infusion. Data from a recent pharmacokinetic cross‐over study revealed that subcutaneously administered DIZ102 and intravenously administered DIZ101 both result in levodopa plasma levels equivalent to those obtained with LCIG. However, although the carbidopa:levodopa ratio is lower in DIZ101 and DIZ102 (1:8) than in LCIG (1:4), they produced nearly four times higher carbidopa levels than LCIG (350–1300 ng/mL vs. 70–480 ng/mL).^9^

Whereas some studies suggest that carbidopa levels higher than those normally achieved with levodopa/carbidopa tablets may be beneficial with respect to levodopa pharmacokinetics,^10^ it has been argued that high levels of carbidopa in blood might lead to enough carbidopa entering the brain to counter the therapeutic effect of levodopa by inhibiting its central conversion to dopamine. While this possibility gains support from animal experiments,^11^, ^12^, ^13^, ^14^ recent clinical studies suggest that oral carbidopa doses up to at least 500 mg/day—doses sometimes used clinically^15^—do not reduce the efficacy of levodopa.^16^, ^17^ Further studies settling this important issue seem warranted.

The subjects in this cross‐over study served as their own controls exposed to the same plasma levodopa concentrations at three different occasions but to either relatively low (when on LCIG) or considerably higher (when on DIZ101 or DIZ102) plasma concentrations of carbidopa. This outcome provides an attractive possibility to investigate the possible negative impact of high plasma carbidopa levels on levodopa efficacy. We analyzed motor symptom evaluations obtained by means of (1) blinded rating of video‐recorded motor function and (2) a wrist‐worn device monitoring bradykinesia, dyskinesia, and tremor (Parkinson KinetiGraph®).^18^

## Subjects/Materials and Methods

### Study Design

Patients with PD having ongoing stable LCIG treatment and Hoehn and Yahr stage <4 during LCIG administration were given LCIG, DIZ101, and DIZ102 using an open‐label cross‐over design. Whereas patient recruitment and end of study safety follow‐up were undertaken at five university neurology clinics in Sweden, the three occasions of 16 h treatment with study medication, blood sampling, and motor function evaluation were all undertaken at a phase I unit (Gothia Forum Clinical Trial Centre at Sahlgrenska University Hospital, Gothenburg, Sweden). Details regarding study design have been presented elsewhere.^9^

### Trial Registration Information

The trial is registered at ClinicalTrials.gov NCT03419806.

### Ethics

The study was conducted in accordance with the International Conference on Harmonization Good Clinical Practice guidelines and the Declaration of Helsinki and approved by the Swedish Ethical Review Authority (658‐17) and the Swedish Medical Products Agency (EudraCT: 2017‐002488‐17). All participants provided written informed consent.

### Randomization and Masking

Patients were allocated to order of treatment with permuted block randomization, a block size of six, and equal distributions of the six possible treatment orders using a pseudorandom generator (SAS Institute, Cary, NC, US). Masking of treatment during video recordings of motor performance was achieved using dummy infusion lines and pumps.

### Procedure

No levodopa or carbidopa administration was allowed for at least 8 h before initiation of trial medication. Between treatment visits, which were separated by at least 3 days, the patients used their regular LCIG treatment.

For the administration of DIZ101 and DIZ102, a concentrated levodopa (20 mg/mL) plus carbidopa (2.5 mg/mL) solution was continuously mixed with the buffer (1:1) using two Braun SPACE Infusion Pumps (B. Braun Melsungen AG, Melsungen, Germany) and a Y‐connector. The resulting concentrations of levodopa and carbidopa in the administered solution hence were 10 and 1.25 mg/mL, respectively. DIZ101 was infused using a peripheral venous catheter in the arm and DIZ102—after a split of the infusion line—using two subcutaneous infusion catheters (Cleo 90; Smiths Medical, MN) at the abdomen. LCIG was delivered using the patient's regular pump (CADD‐Legacy Duodopa; Smith Medical, Minneapolis, MN, USA) and implanted percutaneous transgastric jejunostomy tube.

All treatments were administered as a morning bolus dose followed by an infusion with a fixed flow rate for the rest of the treatment period. The dosing of the three treatments was individualized to match the patient's individual pre‐study LCIG dose as closely as possible taking into account the results of a pilot study indicating that levodopa bioavailability, as compared with DIZ101, is 82% for LCIG and 95% for DIZ102, respectively.^9^ Because of somewhat slower initial levodopa uptake with subcutaneous administration, the bolus dose for DIZ102 was 155% of the hourly dose whereas it was 110% for LCIG and DIZ101. Subsequent changes in flow rate were allowed only if medically necessary.

Pharmacokinetic data showing bioequivalence for the three treatment modalities with respect to plasma levodopa levels, but markedly higher carbidopa levels following administration of DIZ101 and DIZ102, were presented in a previous report, where also tolerability data were provided.^9^

### Rating of Instrumental Tests of Motor Function

At baseline and at 1.5, 5, 6, 7, and 14 h after the onset of drug administration, patients were video‐recorded when performing the following tasks included in the motor examination (part 3) of the Unified Parkinson's Disease Rating Scale (UPDRS): finger tapping (item 23), hand pronation‐supination (item 25), leg movement (item 26), rise from chair (item 27), and gait (item 29).^19^ The recordings were independently rated (0–4) with respect to each item as well as overall bradykinesia (item 31) by two movement disorder specialists who were unaware of the given treatment and order of recording. For each time point, the sum score for these items was used as an overall measure of parkinsonism. Intensity of dyskinesia during the performance of the different UPDRS tasks was rated 0–4 in accordance with the Unified Dyskinesia Rating Scale (UDysRS).^20^ The mean ratings from the two raters were used for further calculations.

### Parkinson's KinetiGraph

The Parkinson's KinetiGraph (PKG) is a wrist‐worn device provided by Global Kinetics (Melbourne, Australia) for the monitoring of bradykinesia, dyskinesia, and tremor in patients with PD.^18^ The device was worn by the patients on the wrist of the most afflicted side from shortly before the infusion started until 24 h had passed. The PKG calculates a bradykinesia (BK) and a dyskinesia (DK) score for every 2 min of measurement resulting in a standard daytime median measure for the time period 09:00–18:00. The fluctuation dyskinesia score (FDS) is a value based on the combined variabilities of BK and DK for the same time period.^21^ In addition, the instrument measures percent time with tremor, again from 09:00–18:00. In the standard PKG report, the BK and DK scores are filtered to exclude bins where the BK score is higher than 80 for more than 50% of the time as this is likely to indicate sleep. However, since patients in the present study were more sedentary due to the study being performed at a phase I unit rather than in freely moving outpatients, applying this filtering resulted in many time points lacking data. In addition to the standard filtered median measures for the time period 09:00–18:00, BK and DK data are therefore also presented without filtering for BK > 80: BK_raw_ and DK_raw_. These scores were aggregated in 30‐min bins, the median score for every bin being used for further analyses. In addition, BK_raw_ and DK_raw_ values were grouped in five time periods: pre‐treatment, 0–2 h, 2–8 h, 8–16 h, and 16–24 h.

### Statistics

Inter‐rater agreement for the assessment of video‐recorded motor function was calculated with Fleiss‐Cohen weighted kappa statistics. The three treatments were compared with respect to UPDRS‐based rating of parkinsonism using repeated measures ANOVA with the factors treatment and time. In a separate analysis, this comparison was repeated but including only subjects who, when treated with DIZ102, displayed plasma mean levels of carbidopa exceeding 700 ng/mL from 2 h after the onset of infusion until this was arrested 14 h later (n = 8). The Greenhouse–Geisser correction was used when sphericity could not be assumed. Possible differences between treatments with respect to filtered standard PKG measures (BK, DK, FDS, PTT) for the 09:00–18:00 period were analyzed using the Friedman's Two‐way Analysis of Variance by Rank, and the same test was also applied for comparing groups with respect to the medians of 30‐min aggregated BK_raw_ and DK_raw_ scores for the five time periods. Correlations between scores from video rating of UPDRS items and the nearest preceding 30 min BK_raw_ or DK_raw_ scores were analyzed with Spearman's rho test. When not otherwise stated, data are presented as group means with standard deviations (SD) or median with interquartile range. Statistical analyses were performed with IBM SPSS Statistics for Windows, Version 28.0 (IBM Corp, Armonk, NY, USA).

## Data Sharing

The anonymized patient data and related clinical trial study documents are not being publicly shared as long as they are part of an ongoing or planned regulatory submission. Anonymized pharmacokinetic and pharmacodynamic data can be provided to qualified researchers after approval of the research proposal.

## Results

### Patient Characteristics

Information on study participants, safety data, and the outcome of the pharmacokinetic analyses have been reported in detail elsewhere.^9^ In short, 20 patients were recruited—8 women and 12 men—with advanced PD and daily LCIG levodopa doses of 641 to 2205 mg as regular treatment. Two patients withdrew from the study after the first treatment (which in both cases was DIZ101).

**Figure 1:**
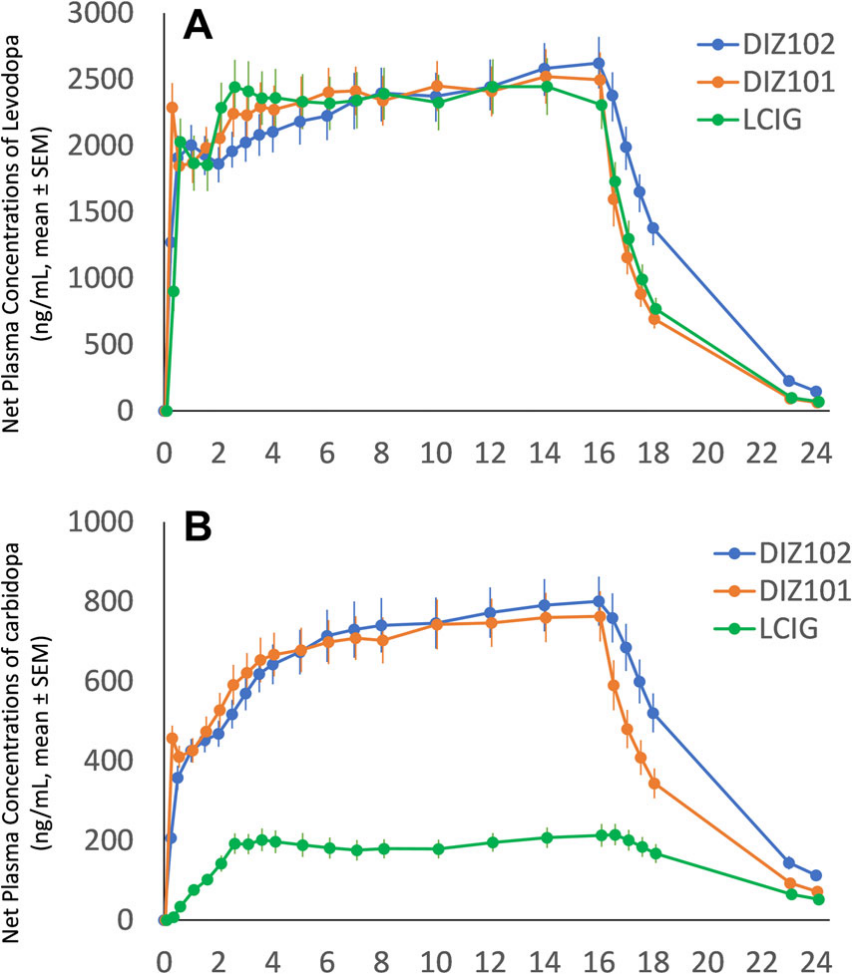
(A) Net plasma concentrations (ng/mL, mean ± SEM) of levodopa (A) and carbidopa (B) before, during 16‐h infusion, and up to 24 h after infusion start with the three treatments DIZ101 (intravenous), DIZ102 (subcutaneous), and LCIG (intestinal). Data from Bergquist et al, Neurology, 2022, doi: 10.1212/WNL.0000000000200804 under Creative Commons license CC BY (licensor American Academy of Neurology, AAN). Graphs from the original publication have been merged into one figure.

Pharmacokinetic data, which were primary effect parameters of this study, have been published previously.^9^ The mean (SD) total levodopa doses of DIZ101, DIZ102, and LCIG over the 16 h infusions in the 18 subjects completing the study were 959 (303), 1019 (321), and 1179 (374), respectively. Plasma mean levels of levodopa (ng/mL) from 2 h to 16 h after the onset of infusion (Fig. 1A) were similar for DIZ101 (mean: 2389, min: 1273, max: 4162), DIZ102 (mean: 2347, range: 1365–4302), and LCIG (mean: 2375, min: 1283, max: 4265). In contrast, mean plasma levels of carbidopa (ng/mL) during the same period (Fig. 1B) were higher for DIZ101 (mean: 711, min: 351, max: 1307) and DIZ102 (mean: 722, min: 410, max: 1313) than for LCIG (mean: 191, min: 73, max: 477).

Carbidopa levels exceeding 700 ng/mL were observed in eight of the subjects when treated with DIZ102 and in seven of these also when treated with DIZ101; this subgroup of eight participants was used in a sensitivity analysis to evaluate if patients with the highest carbidopa levels experienced a reduction in treatment efficacy compared to the response to LCIG in the same subjects. The mean (SD) total levodopa doses of DIZ101, DIZ102, and LCIG over the 16 h infusions in these subjects were 1125 (346), 1199 (361), and 1393 (425), respectively. Mean plasma levels of carbidopa (ng/mL) in this subgroup from 2 h after the start of infusion until this was stopped 14 h later were 901 (min: 683, max: 1307) for DIZ101, 929 (min: 703, max: 1313) for DIZ102, and 245 for LCIG (min: 126, max: 477).

### Video Ratings

**Figure 2:**
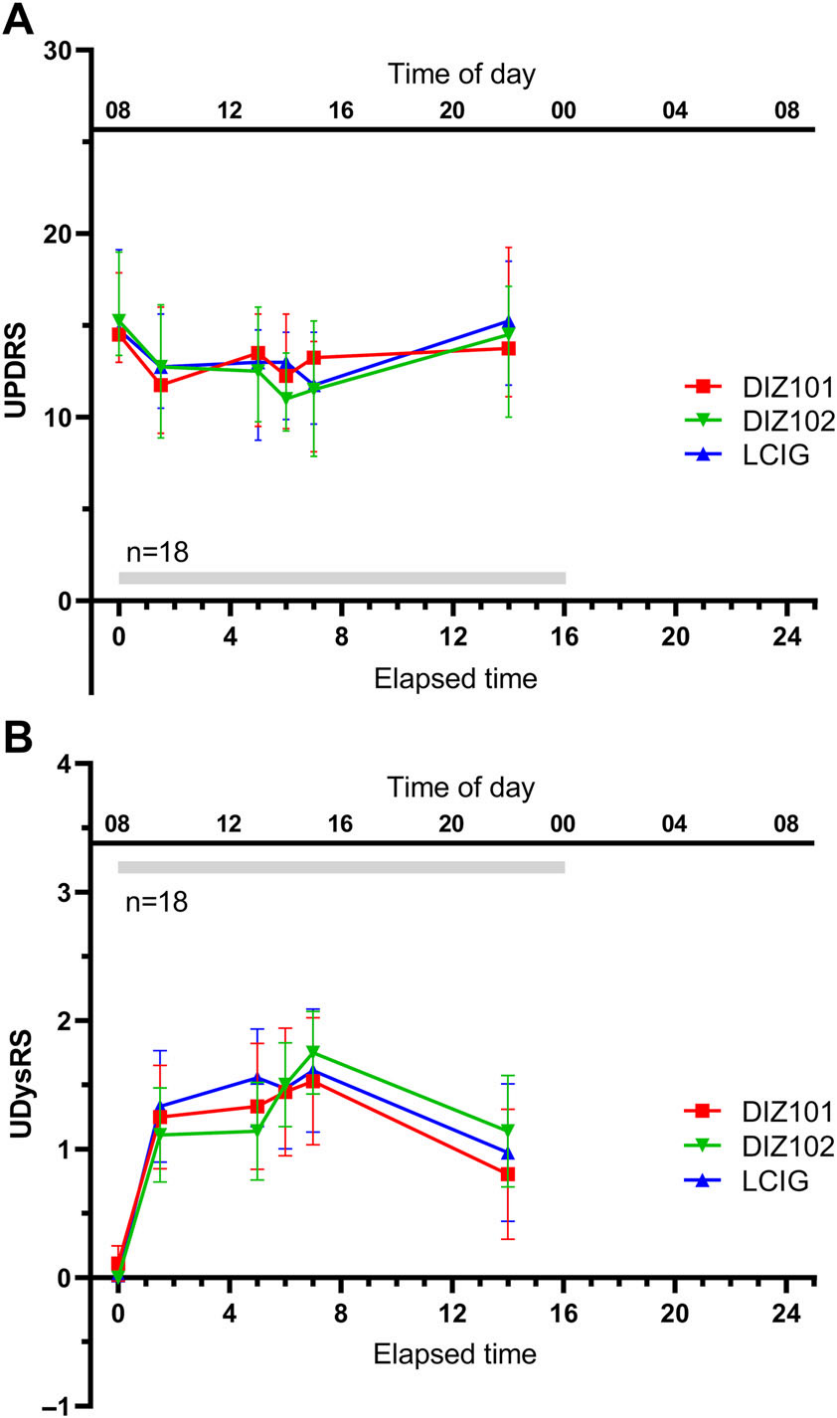
Ratings of video‐recordings during the performance of Unified Parkinson's Disease Rating Scale (UPDRS) items with respect to (A) a subset of UPDRS items reflecting parkinsonism and (B) dyskinesia intensity according to Unified Dyskinesia Rating Scale (UDysRS). The first video recording was performed in the hour before infusion start.

There was substantial interrater agreement between raters for UDysRS with a Kappa value of 0.74 (95% CI 0.69–0.79) and moderate agreement for UPDRS with a Kappa value of 0.46 (95% CI 0.39–0.42). The UPDRS subscores were reduced by 2.5 (LCIG), 2.6 (DIZ101), and 3.3 (DIZ102) points, respectively, between baseline and the first assessment at 1.5 h after start of treatment. UPDRS ratings remained significantly lower until 7 h after treatment start (Fig. 2A). The mean UPDRS subscore ratings of video recordings hence showed a significant effect of the factor time (*F*(2.57, 41.54) = 14.82, *P* < 0.0005) but no significant effect of the factor treatment (*F*(1.61, 25.74) = 0.30, *P* = 0.7) and no interaction between time and treatment (*F*(3.87, 61.96) = 0.580, *P* = 0.7). UPDRS subscores in the subpopulation of subjects (n = 8) displaying carbidopa mean levels (≥700 ng/mL) during infusion of DIZ102 (Fig. S1) also displayed a significant effect of time (*F*(2.05, 14.35) = 6.28, *P* < 0.011) but no significant effect of treatment (*F*(1.36, 9.53) = 2.03, *P* = 0.19) and no interaction between time and treatment (*F*(4.62, 32.34) = 1.19, *P* = 0.34).

The maximum UDysRS ratings were approximately 1.5 and observed 5–7 h after start of infusion with all treatments (Fig. 2B). Again, there was a significant effect of time (*F*(2.50, 40.03) = 26.76, *P* < 0.0005) but not of treatment (*F*(1.72, 27.57) = 0.316, *P* = 0.7) and no significant time × treatment interaction (*F*(4.40, 70.48) = 1.57, *P* = 0.2) (Fig. 2B).

### Objective Accelerometry Measurements of Spontaneous Movements

For technical reasons, PKG measurements were missing from one patient during treatment with DIZ101 and from another subject during treatment with DIZ102; PKG data were hence available for all treatments from 16 of the 18 participants. The summary PKG measures for these 16 subjects with respect to BK score, DK score, FDS, and PTT did not display different distributions with the three treatments (Table 1). For all treatments, the DK scores as well as FDS were within suggested limits for controlled dyskinesia (DK <7 and FDS <10.8). The BK scores for all treatments were slightly over the limit (≤BK 25) for controlled bradykinesia.^22^

**TABLE 1:** Standard summary measures PKG, median (IQR)

Treatment	BK50_09‐18_	DK50_09‐18_	FDS_09‐18_	PTT_09‐18_
DIZ101	28.1 (8.1)	0.9 (2.3)	8.1 (3.1)	0.4 (1.1)%
DIZ102	25.8 (9.2)	2.3 (2.4)	8.4 (4.4)	0.4 (0.5)%
LCIG	28.6 (12.5)	1.1 (4.7)	9.3 (3.1)	0.7 (0.9)%
*P*‐value^a^	0.538	0.180	0.059	0.270

^a^ Friedman's two‐way analysis of variance by rank.

**Figure 3:**
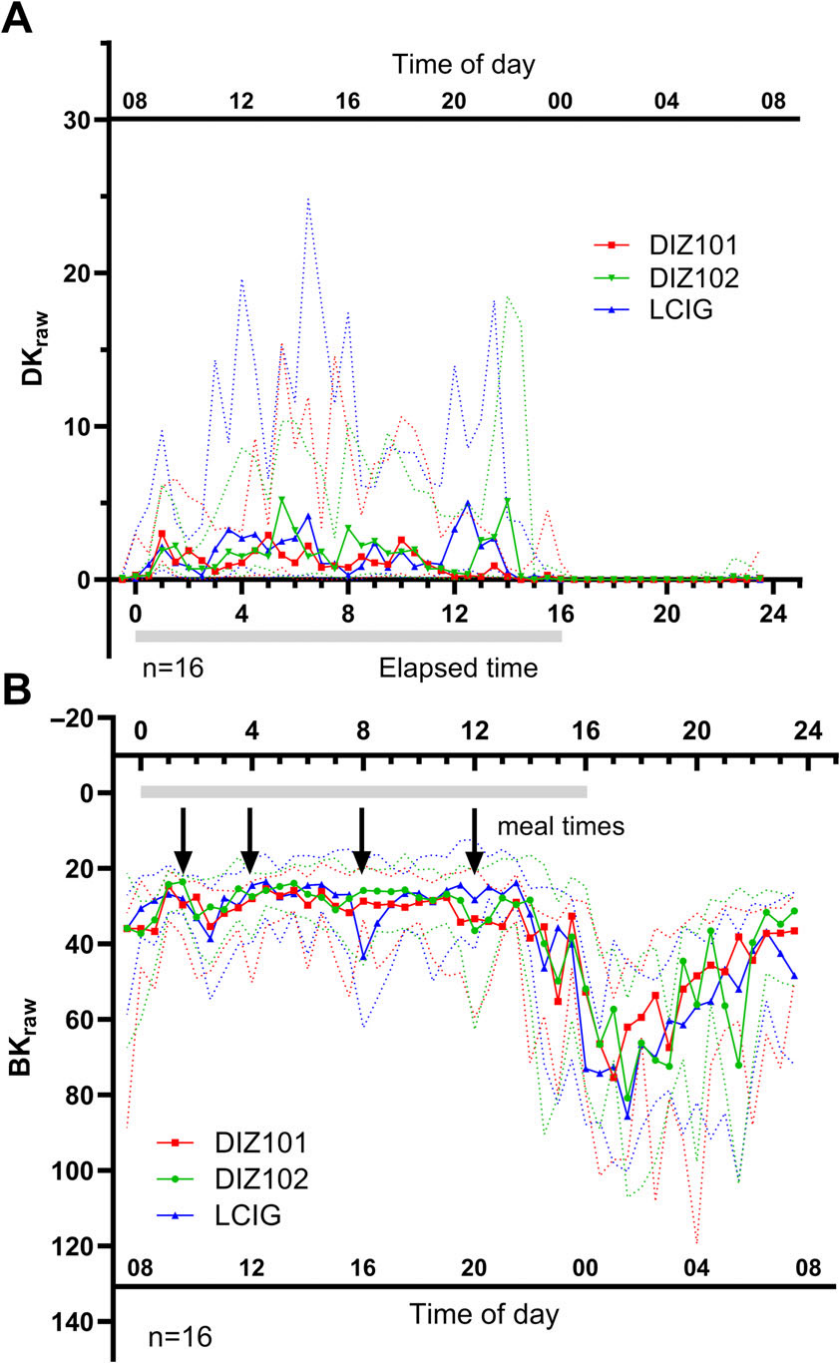
Unfiltered (A) dyskinesia (DK_raw_) and (B) bradykinesia (BK_raw_) scores obtained with the Parkinson KinetiGraph. Scores were aggregated in 30‐min bins which include up to 15 2‐min scores; the median score for each bin is reported. Data are presented as median (continuous line) and lower/upper quartiles (dashed line). BK scores over 80, which are normally filtered out, were included (see Methods). Arrows indicate time points when patients were provided with breakfast, lunch, dinner, and an evening snack. The horizonal bar indicates time of drug infusion.

**Figure 4:**
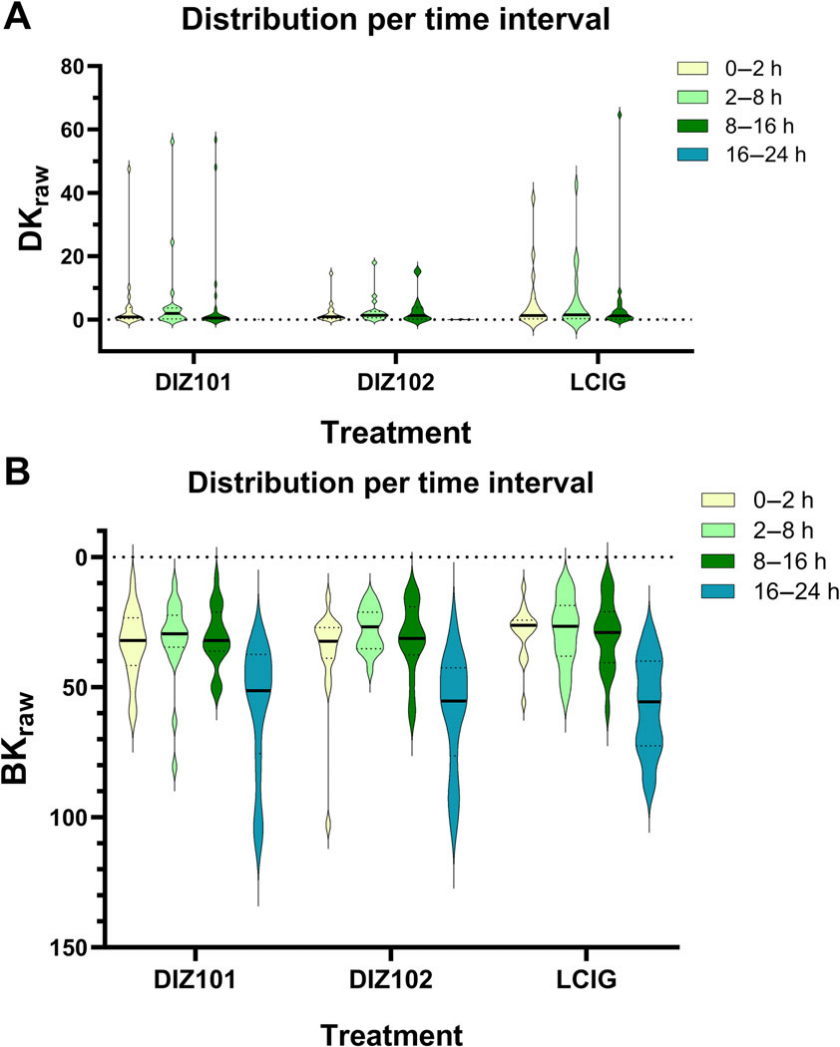
Distribution of DK_raw_ (A) and BK_raw_ (B) scores during the different pre‐defined time periods illustrated with violin diagrams.

The unfiltered BK_raw_ and DK_raw_ PKG scores aggregated in 30 min‐bins over the treatment days are presented in Figure 3. Analyses of prespecified time periods during and after the infusions did not demonstrate differences in distributions of BK_raw_ or DK_raw_ between the three treatments for any of the time periods (Table 2, Fig. 4). Including patients for whom measurements from one of the treatments were missing, did not change the outcome (data not shown).

**TABLE 2:** Median (IQR) BK_raw_ and DK_raw_ measures for pre‐specified time periods

Treatment	0–2 h	2–8 h	8–16 h	16–24 h	1‐16 h
BK_raw_					
DIZ101	32.0 (18.6)	29.8 (11.2)	33.5 (9.4)	52.5 (45.4)	30.9 (10.3)
DIZ102	30.4 (12.7)	28.0 (14.7)	31.2 (23.8)	55.0 (37.9)	29.8 (14.8)
LCIG	26.1 (5.3)	29.0 (19.2)	29.4 (19.8)	60.0 (33.3)	29.6 (17.4)
*P*‐value^a^	0.472	0.118	0.751	0.926	0.472
DK_raw_					
DIZ101	0.8 (3.3)	1.0 (3.1)	0.3 (0.9)	0.0 (0.0)	0.8 (1.9)
DIZ102	1.0 (3.0)	1.3 (1.9)	1.3 (4.3)	0.0 (0.0)	1.3 (2.8)
LCIG	1.9 (4.7)	0.9 (6.0)	0.9 (2.7)	0.0 (0.0)	0.8 (4.6)
*P*‐value^a^	0.779	0.423	0.282	0.943	0.426

^a^ Friedman's two‐way analysis of variance by rank.

### Correlation between Video Ratings and Accelerometry Data

There was a positive correlation between the median UPDRS items total score and the preceeding BK_raw_ value (*r* = 0.60, *P* < 0.001) and between median UDysRS scores and the preceeding DK_raw_ value (0.64, *P* < 0.001).

## Discussion

Neither the blinded clinical video ratings of parkinsonism and dyskinesia nor the objective PKG recordings revealed significant and consistent differences between DIZ101, DIZ102, and LCIG with respect to symptom reduction or dyskinesia. While motor symptoms were not a primary effect parameter, and the trial was not designed to show non‐inferiority between the different treatments in this regard, the lack of even a tendency of poorer outcome with DIZ102 suggests that this less invasive treatment, if clinically implemented, may be as effective as LCIG for the management of motor fluctuations in patients with PD.

The optimal ratio of carbidopa to levodopa for oral treatment of PD remains a matter of debate. While a 1:4 ratio is most common, some studies suggest that higher amounts of carbidopa might translate into more favorable levodopa pharmacokinetics.^10^ On the other hand, there are animal data indicating that high doses of carbidopa may lead to a significant amount of the enzyme inhibitor entering the brain, thereby reducing central dopamine formation.^11^, ^12^, ^13^, ^14^ Although DIZ101 and DIZ102 display a lower ratio of carbidopa vs levodopa (1:8) than does LCIG (1:4), the bioavailability of carbidopa being complete with DIZ101 and DIZ102, but less than 20% with LCIG, results in roughly four times higher carbidopa levels during treatment with the former products.^9^ Given that the bioavailability of orally administered carbidopa appears to be between 30 and 40%,^23^ the levels following DIZ101 and DIZ102 are likely to be higher also than those obtained with corresponding doses of oral preparations containing carbidopa and levodopa with a 1:4 ratio. Since the patients in the present trial served as their own controls in situations where levodopa levels were the same, but carbidopa levels were markedly different, the study provided an excellent possibility to investigate if plasma carbidopa levels higher than those usually obtained with oral treatment may negatively impact the motor response to a given amount of levodopa. Our observation of similar motor treatment efficacy in the three treatment groups, with no tendency for more bradykinesia in subjects receiving DIZ101 or DIZ102, hence provides robust support for the conclusion that carbidopa levels of the magnitude obtained with LCIG101 and LCIG102 do not counter the efficacy of levodopa.

The participants in the present trial were dosed in accordance with their levodopa requirement; hence both dosing and plasma levels of carbidopa (and levodopa) varied to a considerable extent. To exclude the possibility that those with the highest plasma carbidopa levels might display a reduced response not observed in the entire group, a sensitivity analysis in the subgroup of subjects (n = 8) displaying mean carbidopa levels above 700 ng/mL (min: 706 ng/mL; max: 1313 mg/mL) when subjected to DIZ102 was undertaken. Also in this subgroup, there were, however, no indications of a reduced treatment response with DIZ102 or DIZ101 as compared to LCIG (Fig. S1).

Our findings are in agreement with a recent paper by Trenkwalder and co‐workers according to which an increase in carbidopa exposure by 2.5–4 times in patients on oral levodopa/carbidopa in conjunction with a COMT inhibitor resulted in a reduction (rather than an increase) in off‐time.^16^ Likewise, Brod and co‐workers have reported that increasing the daily oral dose of carbidopa from 75 to 450 mg did not reduce the efficacy of levodopa but increased it in a majority of the participants.^17^

This study did not address the possible pharmacokinetic benefit of the relatively high plasma carbidopa levels obtained with DIZ101 and DIZ102 with respect to the inhibition of the peripheral conversion of levodopa to dopamine. It is, however, likely that higher levels of carbidopa in blood may be required with parenteral administration as compared to the situation with LCIG and oral treatment, where carbidopa is administered directly into the intestines, hence producing high concentrations at the sites where the conversion of levodopa to dopamine to a great extent takes place, ie, the intestines and the liver, without these concentrations being reflected by the levels in blood. A previous report regarding another levodopa plus carbidopa product aimed for subcutaneous administration, ND0612, suggests that the carbidopa vs levodopa ratio 1:8, at hand in both DIZ101, DIZ102, and ND0612, is adequate from the perspective of optimizing levodopa pharmacokinetics.^24^

In the present group of patients with PD, who were all on regular treatment with LCIG when included, all three treatments resulted in a satisfactory motor response. The tendency for increased bradykinesia throughout the day with all treatments, despite stable levodopa levels, is a well‐established phenomenon observed also in previous LCIG studies.^25^

There are few independent studies where objective accelerometry data obtained with commercial wearables such as the PKG have been compared to symptom rating undertaken by clinicians. The present study provides near simultaneous video‐based symptom rating and PKG registration combined with levodopa plasma concentrations in patients with PD displaying motor fluctuations. Although the setting was different in the present study as compared to when wearables have been used in outpatients, the correlation between PKG data from the most recent 30 min and clinical symptom rating was similar to what has previously been described when patient‐rated symptomatology has been compared to median BK and DK data during a 10‐day period.^26^

Some limitations are worth mentioning. The data were collected as secondary outcomes in an acute pharmacokinetic study which was not designed to demonstrate non‐inferiority regarding motor efficacy; its design made it difficult to include evaluation of important non‐motor symptoms like sleep; the included subjects may be too few to detect subtle differences in motor efficacy between the three treatments. However, the lack of even a tendency of poorer outcome with the treatments associated with markedly higher plasma levels of carbidopa, ie, DIZ101 and DIZ102, argues against the possibility that the absence of significant superiority of LCIG in terms of motor efficacy be explained by insufficient statistical power. Second, the therapeutic response observed during a 16 h period of administration at a phase I unit does not necessarily reflect the response upon long term administration to outpatients.

In conclusion, the present results make it unlikely that the response to DIZ101 and DIZ102 should differ markedly from that obtained with LCIG in case the former treatments should be clinically implemented. Moreover, in line with other recent reports,^16^, ^17^ they suggest that carbidopa doses causing markedly higher blood carbidopa levels than those obtained with conventional oral carbidopa plus levodopa treatment (with a 1:4 ratio) are not likely to reduce levodopa efficacy.

## Author Roles

(1) Research project: A. Conception, B. Organization, C. Execution, D. Review and critique of design; (2) Statistical Analysis: A. Design, B. Execution, C. Review and Critique; (3) Manuscript: A. Writing of the first draft, B. Review and Critique.

F.B.: 1A, 1B, 1C, 2A, 2B, 3A, 3B

M.E.: 1A, 1B, 1D, 2C, 3B

A.J., F.L., D.N., P.O., P.S.: 1C, 1D, 3B

N.D.: 1A, 1D, 2C, 3B

E.E.: 1A, 1B, 2A, 2C, 3A, 3B

All authors approved the final version of the manuscript. All authors confirm that they had full access to all the data in the study and accept responsibility to submit for publication.

## Disclosures

**Ethical Compliance Statement:** The study was approved by the Swedish Ethical Review Authority (658‐17). All participants provided written informed consent. We confirm that we have read the Journal's position on issues involved in ethical publication and affirm that this work is consistent with those guidelines.

**Funding Sources and Conflicts of Interest:** This study was funded by the Swedish Research Council (Grant No. 2014‐07298), the Swedish state under the agreement between the Swedish government and the county councils (ALF‐agreement Grant Nos. 73220 and 77340), the Parkinson Research Foundation at the Linköping University, and Dizlin Pharmaceuticals AB. Bergquist is a stock option holder of Dizlin Pharmaceuticals AB and a principal investigator in Abbvie trials with ABBV‐951. Ehrnebo is a coinventor (but not owner) of a patent regarding DIZ101 and DIZ102 and the founder and owner of Ehrnebo Development AB that holds equity in Dizlin Pharmaceuticals AB. Eriksson is a coinventor (but not owner) of a patent regarding DIZ101 and DIZ102, and is a stock and stock option holder of Dizlin Pharmaceuticals AB. Dizdar is a coinventor (but not owner) of a patent regarding DIZ101 and DIZ102 and stock and stock option holder of Dizlin Pharmaceuticals AB. Lundin, Svenningson and Johansson report no disclosures relevant to the manuscript.

**Financial Disclosures for the Previous 12 Months:** PO has received fees for lectures and advice from AbbVie and Britannia. DN has received consulting fees from Britannia, Stada, NordicInfu Care, and NeuroDerm and lecture honoraria from AbbVie. The other authors have no additional disclosures to report.

## Supporting information


**Figure S1.** Ratings of video‐recordings of during the performance of Unified Parkinson's Disease Rating Scale (UPDRS) items with respect to a subset of UPDRS items reflecting parkinsonism in the subgroup of subjects displaying the highest blood carbidopa levels when treated with DIZ102 (n = 8).

## Data Availability

The anonymized patient data and related clinical trial study documents are not being publicly shared as long as they are part of an ongoing or planned regulatory submission. Anonymised pharmacokinetic and pharmacodynamic data can be provided to qualified researchers after approval of the research proposal.
