# Supplementary material for: Motor Efficacy of Subcutaneous DIZ102, Intravenous DIZ101 or Intestinal Levodopa/Carbidopa Infusion
Source: Mov Disord Clin Pract. 2024 Jun 24;11(9):1095–102. doi: 10.1002/mdc3.14138 (PMC11452806; doi:10.1002/mdc3.14138)
Supplement: Supplementary file 1 — Figure S1. Ratings of video‐recordings of during the performance of Unified Parkinson's Disease Rating Scale (UPDRS) items with respect to a subset of UPDRS items reflecting parkinsonism in the subgroup of subjects displaying the highest blood carbidopa levels when treated with DIZ102 (n = 8). [file MDC3-11-1095-s001.docx]

**Supplemental material**


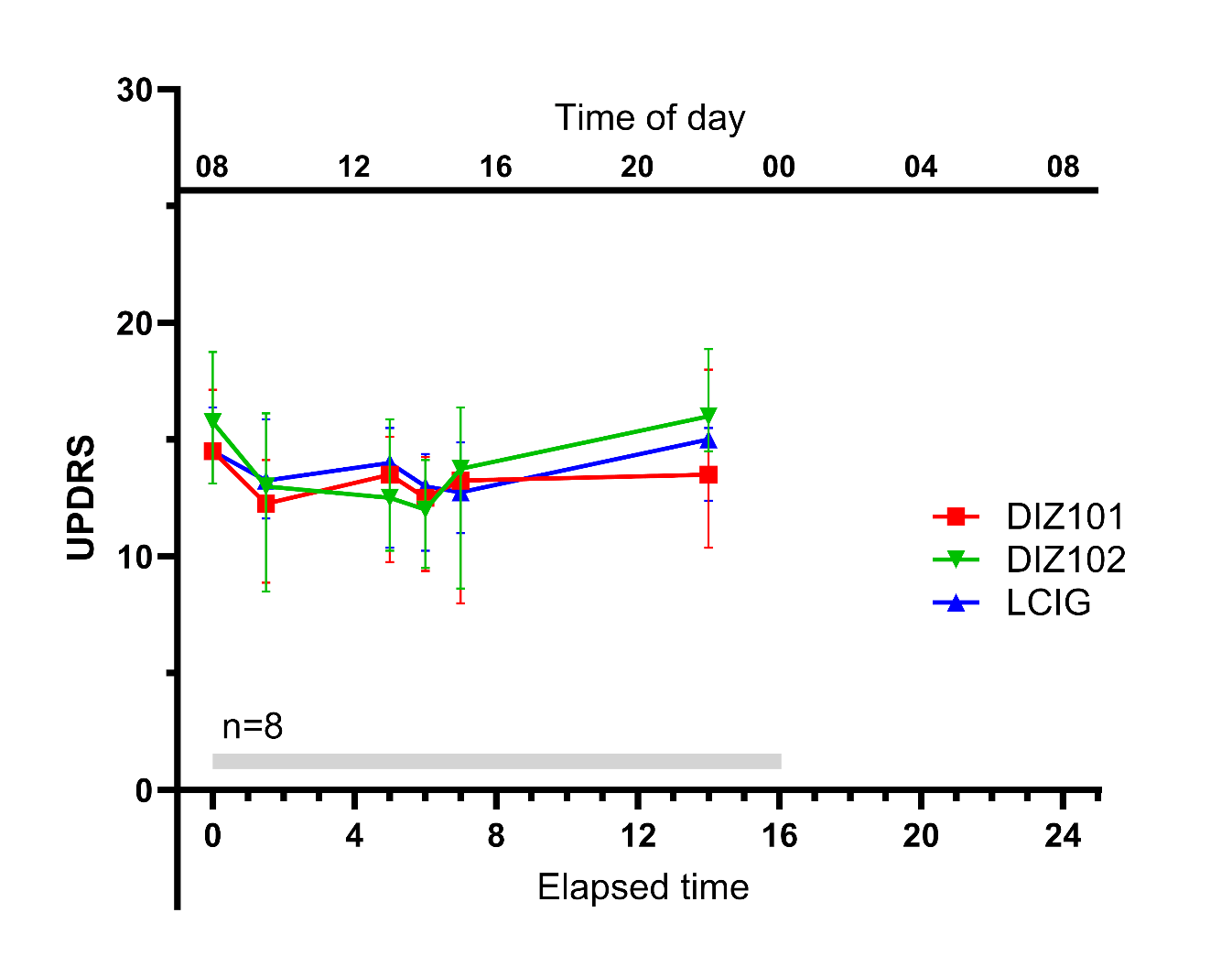


Figure S1.

Ratings of video-recordings of during the performance of UPDRS items with respect to a subset of UPDRS items reflecting parkinsonism in the subgroup of subjects displaying the highest blood carbidopa levels when treated with DIZ102 (n=8).
